# Protein Kinase D3 promotes the cell proliferation by activating the ERK1/c‐MYC axis in breast cancer

**DOI:** 10.1111/jcmm.14772

**Published:** 2020-01-16

**Authors:** Yan Liu, Hang Song, Shiyi Yu, Kuo‐Hsiang Huang, Xinxing Ma, Yehui Zhou, Shuang Yu, Jingzhong Zhang, Liming Chen

**Affiliations:** ^1^ The Key Laboratory of Bio‐Medical Diagnostics Suzhou Institute of Biomedical Engineering and Technology Chinese Academy of Sciences Suzhou China; ^2^ Changchun Institute of Optics, Fine Mechanics and Physics Chinese Academy of Sciences Changchun China; ^3^ Jiangsu Key Laboratory for Molecular and Medical Biotechnology College of Life Science Nanjing Normal University Nanjing China; ^4^ The Key Laboratory of Developmental Genes and Human Disease Ministry of Education, Institute of Life Science Southeast University Nanjing China; ^5^ Shanghai Children’s Medical Center School of Medicine Shanghai Jiaotong University Shanghai China; ^6^ The First Affiliated Hospital of Soochow University Soochow University Suzhou China; ^7^ Xuzhou Medical University Xuzhou China; ^8^ Tianjin Guokeyigong Science and Technology Development Company Limited Tianjin China

**Keywords:** breast cancer, c‐MYC, ERK1, Protein Kinase D3

## Abstract

Breast cancer is the second leading death cause of cancer death for all women. Previous study suggested that Protein Kinase D3 (PRKD3) was involved in breast cancer progression. In addition, the protein level of PRKD3 in triple‐negative breast adenocarcinoma was higher than that in normal breast tissue. However, the oncogenic mechanisms of PRKD3 in breast cancer is not fully investigated. Multi‐omic data showed that ERK1/c‐MYC axis was identified as a major pivot in PRKD3‐mediated downstream pathways. Our study provided the evidence to support that the PRKD3/ERK1/c‐MYC pathway play an important role in breast cancer progression. We found that knocking out PRKD3 by performing CRISPR/Cas9 genome engineering technology suppressed phosphorylation of both ERK1 and c‐MYC but did not down‐regulate ERK1/2 expression or phosphorylation of ERK2. The inhibition of ERK1 and c‐MYC phosphorylation further led to the lower protein level of c‐MYC and then reduced the expression of the c‐MYC target genes in breast cancer cells. We also found that loss of PRKD3 reduced the rate of the cell proliferation in vitro and tumour growth in vivo, whereas ectopic (over)expression of PRKD3, ERK1 or c‐MYC in the PRKD3‐knockout breast cells reverse the suppression of the cell proliferation and tumour growth. Collectively, our data strongly suggested that PRKD3 likely promote the cell proliferation in the breast cancer cells by activating ERK1‐c‐MYC axis.

## 1 INTRODUCTION

Breast cancer is one of the common diagnosed adenocarcinoma and the second leading cause of cancer death in women.^1^ The expense associated with breast cancer treatment and the follow‐up care system can be a financial burden for individuals, their families, or even health organization of their countries. Therefore, the pathological mechanism of the breast cancer should be further investigated for developing more efficient diagnosis and therapies.

Protein Kinase D3 has been implicated in a variety of functions in broad ranges of cancer types.^2^, ^3^, ^4^, ^5^, ^6^, ^7^ Additionally, PRKD3 has been suggested to be up‐regulated and involved in mediating the survival, differentiation, migration and proliferation of the triple‐negative breast cancer cells.^8^, ^9^, ^10^ Diacylglycerol (DAG) has been found to bind to the cysteine‐rich domain of PRKD3 and played important roles in PRKD3 activation via PKC signalling.^11^ However, the downstream pathway of PRKD3 is not well‐understood. The integrated phosphoproteomic and transcriptomic analysis in the previous studies showed that PRKD3 may play important roles in a variety of the cancer‐related pathways. ERK1/c‐MYC axis was identified as a major pivot in PRKD3‐regulated pathways in tumour cells.^2^, ^12^ However, the interaction mechanisms among PRKD3, ERK1 and c‐MYC in cancer cells has not been well‐investigated since only one prostate cancer study suggested PRKD3‐activated ERK1/2 is involved in tumour growth.^13^

The extracellular signal‐regulated kinase 1/2 (ERK1/2) can be activated and/ or up‐regulated by upstream kinases, transcription factors and/ or differentiation factor.^14^, ^15^, ^16^ Activated ERK1/2 translocates to the cell nucleus and then phosphorylates a series of transcription factors, such as c‐MYC, FOS, FRA1 and EGR1.^17^, ^18^, ^19^, ^20^, ^21^, ^22^, ^23^ These ERK1/2‐activated downstream factors further regulate many cellular functions, including proliferation, differentiation and transformation.^24^, ^25^, ^26^ In mammals, the identity of ERK1 and ERK2 sequences is 84%, but ERK1 has two more amino acids (a.a.) at its C‐terminus and 17 more a.a. at N‐terminus than ERK2.^27^ Most previous studies had indicated that the functions of ERK1 and ERK2 were redundant, and they usually worked together as a fundamental unit to regulate cellular functions in organisms.^28^ Recently, more and more studies have been found to support that ERK1 and ERK2 can function independently.^29^, ^30^, ^31^, ^32^, ^33^, ^34^, ^35^ It has been reported that c‐MYC was a substrate of ERK, which phosphorylated the Ser62 of c‐MYC and then stabilized c‐MYC.^36^, ^37^ As a transcription factor, c‐MYC has been reported to be involved in a wide variety of carcinogenesis process such as tumour growth and angiogenesis.^38^, ^39^, ^40^, ^41^, ^42^ In addition, it also plays an important role in proliferation, metastasis and senescence in breast cancer cells.^43^, ^44^, ^45^, ^46^ However, it is unclear whether PRKD3 promote breast cancer progression via ERK1/c‐MYC axis.

Our current data showed that knocking out PRKD3 led to the reduction of p‐ERK1, p‐c‐MYC (Ser62), total c‐MYC and the down‐regulated expression of c‐MYC target genes in breast cancer cells. On the contrary, p‐ERK2 and total amount of ERK2 were not reduced in the PRKD3‐knockout cells. In addition, the proliferation of the breast cancer cells and the tumour growth in xenograft mouse models were promoted by both the PRKD3 overexpression and the ERK1/c‐MYC axis activation. In short, our study indicated that PRKD3 likely promoted the proliferation of breast cancer cells by activating ERK1/c‐MYC axis, but not ERK2‐mediated pathways.

## 2 MATERIALS AND METHODS

### 2.1 Cell lines, clone screening and cell culture

The parental breast cancer cell lines, MDA‐MB‐468 and MDA‐MB‐231, were obtained from American Type Culture Collection (ATCC) within 6 months. The PRKD3‐knockout MDA‐MB‐468 and MDA‐MB‐231 cell lines were generated using CRISPR/Cas9 system. The PRKD3‐knockout breast cancer cells were screened with limiting dilutions assays and confirmed by performing Western blotting. All the breast cancer cells were cultured in dulbecco's modified eagle medium supplemented with 1% penicillin‐streptomycin solution and 10% foetal bovine serum.

### 2.2 Transfection

The plasmids were used in the current study: pCMV‐PRKD3‐Flag, pCDNA‐ERK1‐HA and pCDNA‐c‐MYC‐His (Sino Biological Inc). Plasmids were transfected into breast cancer cells with Lipofectamine^®^ 3000 (Invitrogen).

### 2.3 Western blotting

Western blotting was carried out by transfering whole cell protein from 10% SDS‐PAGE onto the PVDF membranes (Millipore) and incubating the membranes with the primary and secondary antibodies. Primary antibodies against PRKD3, p‐ERK1/2 (Thr202/Tyr204), ERK1/2, p‐c‐MYC (Ser62) and c‐MYC were purchased from Cell Signalling Technology. β‐actin primary antibody, Anti‐rabbit and antimouse secondary antibody were purchased from Santa Cruz Biotechnology.

### 2.4 Real‐time RT‐PCR

Total RNAs were extracted from breast cancer cells by using RNeasy kit (Qiagen), and synthesized cDNA was performed by using PrimeScript RT reagent kit (TaKaRa). The primers are listed in Table S1.

### 2.5 Immunofluorescence staining

Breast cancer cells were cultured on glass slides in 24‐well plates. The breast cancer cells were washed with PBS in whole procedure. The cells were fixed with 4% paraformaldehyde (PFA) for more than 30 minutes at room temperature (RT) and then permeabilized/ blocked with PBS containing 0.1% Triton X‐100 /1% BSA for about 1 hour at RT. The primary antibodies (α‐PRKD3, α‐p‐ERK1/2(Thr202/Tyr204), α‐ERK1/2, α‐p‐c‐MYC (Ser62) and α‐c‐MYC antibodies), and the secondary antibodies (Alexa Fluor 555‐conjugated and 488‐conjugated secondary antibody) were used to define the specific protein location. DAPI (Solarbio) was used to locate the nuclei.

### 2.6 Cell proliferation assay

CCK‐8 kit (Dojindo Laboratories) was used to measure breast cancer proliferation according to the protocol recommended by manufacturer. The Breast cancer cell lines were plated out at the same confluence in 96‐well dishes and grown at 37°C for 3 days. CCK‐8 solution was added into each well for 3 hours, then absorbance at 450 nm was measured.

### 2.7 Xenografted tumour formation assay

Breast cancer cells (5 × 10^6^) were injected into the mammary fat pad of female athymeic nude mice. The width and length of breast cancers were measured weekly by using Vernier caliper, and the breast tumour volume was calculated using the formula: 1/2 × (*l*) × (*w*)^2^ [*l*:length; *w*:width].

### 2.8 Human breast tissue process

Human breast cancer tissue specimens were obtained from the first affiliated hospital of Soochow university. After excision, all the human breast tumours were off‐handedly frozen at −80ºC. The RNA in these tissues were extracted, reversely transcripted and quantified by using the kits mentioned in section 2.4. All the patients involved in this study have been informed about their tissue usage in this study and signed the consent letter. This research was performed with the approval of Suzhou Institute of Biomedical Engineering and Technology's medical ethics committee. Information of breast cancer samples are listed in Table S2.

## 3 RESULTS

### 3.1 Generation of PRKD3 gene knockout breast cancer cell lines

**Figure 1:**
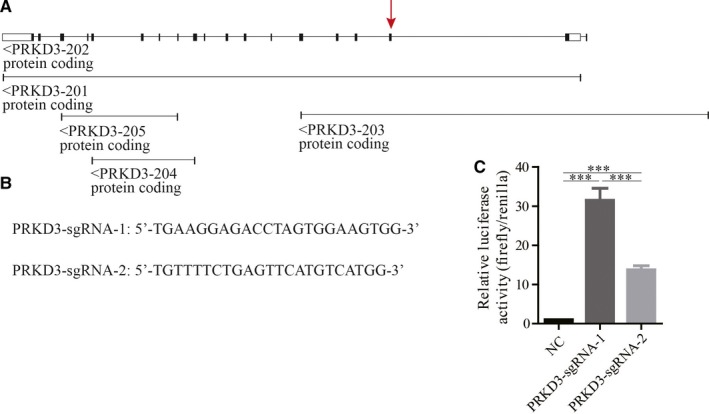
A CRISPR/CAS9 knockout strategy for ablation of the PRKD3 expression. A, The human PRKD3 gene structure and the five pre‐mRNA transcripts encoded the PRKD3 isoforms were shown. The arrow indicated the sgRNA‐targeted exon. B, The two sequences of the PRKD3 sgRNAs. C, The luciferase‐based SSA assay was used to detect the two sgRNA‐guided Cas9 activity. Data represent the mean ± SEM from three biological replicates (n = 3). ***P < .001 by *t* test

Our previous studies have suggested that PRKD3 was overexpressed in triple‐negative breast cancer cell lines (MDA‐MB‐468, MDA‐MB‐231).^2^, ^12^ In order to study PRKD3 signalling pathway in breast cancer, CRISPR/Cas9 technology was applied for knocking out PRKD3 in the cells. It has been reported that five protein isoforms were encoded for the human PRKD3 gene; however, only three of the isoforms contained the kinase domains (Figure 1A). Therefore, we designed the two single‐guide RNAs (sgRNAs) to knock out the three ‘active’ isoforms of PRKD3 by targeting the specific PRKD3 exons (Figure 1B). Luciferase SSA recombination assay showed that the luciferase activity of the lysates extracted from the cell transfected with either one of the two CAS9/gRNAs was at least 10‐fold higher than the one of the control lysate (Figure 1C). Since the luciferase activity of the lysate extracted from the PRKD3‐sgRNA‐1‐edited cells was higher than the one from the PRKD3‐sgRNA‐2‐edited cells, we decided to use PRKD3‐sgRNA‐1 to knockout PRKD3 gene in the two breast cancer cell lines.

### 3.2 Loss of PRKD3 suppresses phosphorylation of ERK1 and c‐MYC

**Figure 2:**
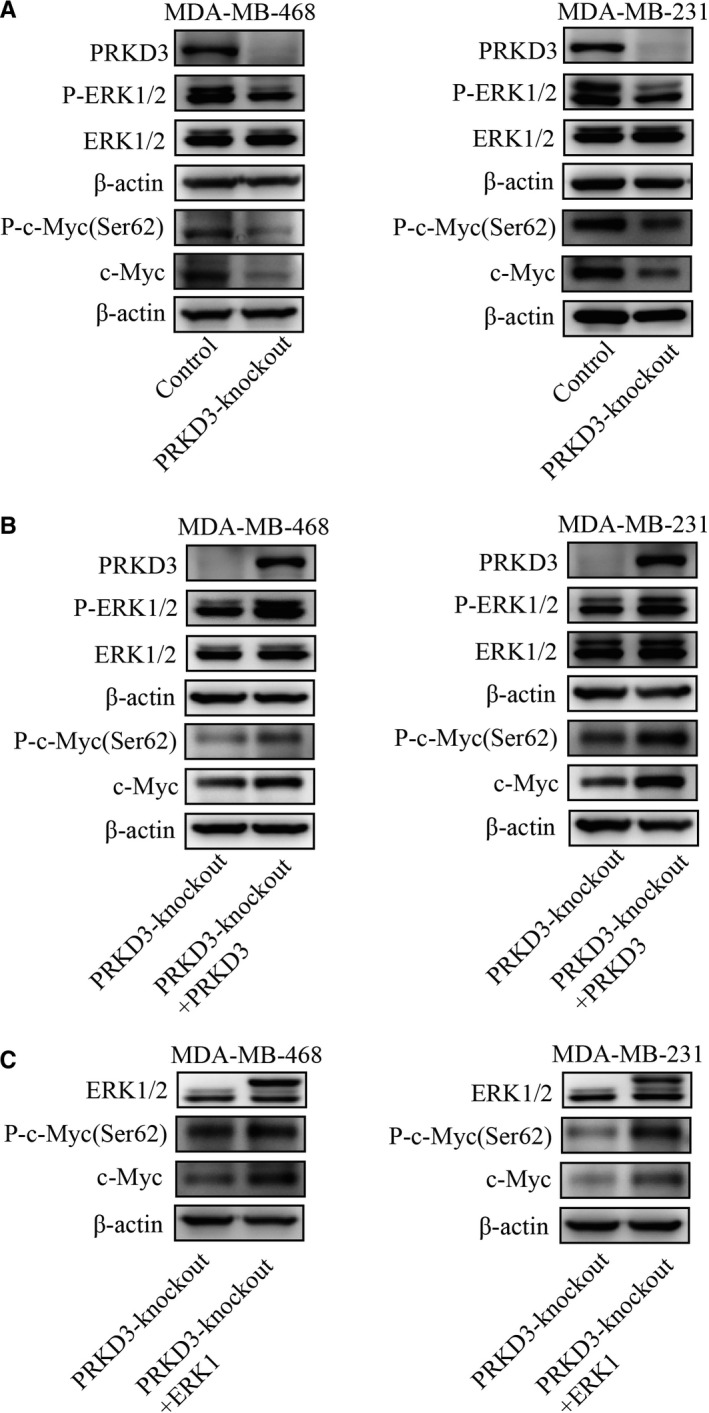
Western blot analysis showed changes in the protein levels among PRKD3, (p‐)ERK1/2 and (p‐)c‐MYC. A, The protein levels of p‐ERK1 (Thr202/Tyr204), p‐c‐MYC (Ser62) and c‐MYC in the PRKD3‐knockout breast cancer cell lines were lower than the ones of these proteins in the parental cell lines (MDA‐MB‐468 and MDA‐MB‐231). B, Ectopic (over)expression of PRKD3 or (C) ERK1 in the PRKD3‐knockout cells led to the increased protein levels of (p‐)c ‐MYC(Ser62)

In order to confirm that PRKD3 activated ERK1/c‐MYC axis in the breast cancer cells, we analysed the amounts of the phosphorylated and total ERK1/2 or c‐MYC by performing Western blotting. We found that the amounts of p‐ERK1 (Thr202/Tyr204), p‐c‐MYC (Ser62), c‐MYC in the PRKD3‐knockout MDA‐MB‐468 and MDA‐MB‐231 cell lines were lower than the ones in the parental cell lines. However, the amounts of p‐ERK2 (Thr202/Tyr204) and ERK1/2 in the breast cancer cells were not reduced in the PRKD3‐knockout cells (Figure 2A). Additionally, ectopic expression of PRKD3 in the PRKD3‐knockout breast cancer cell lines led to the increased amount of p‐ERK1(Thr202/Tyr204), p‐c‐MYC, and c‐MYC (Figure 2B). Furthermore, overexpression of ERK1 in the PRKD3‐knockout cells is sufficient to increase the amounts of p‐c‐MYC(Ser62) and c‐MYC (Figure 2C).

**Figure 3:**
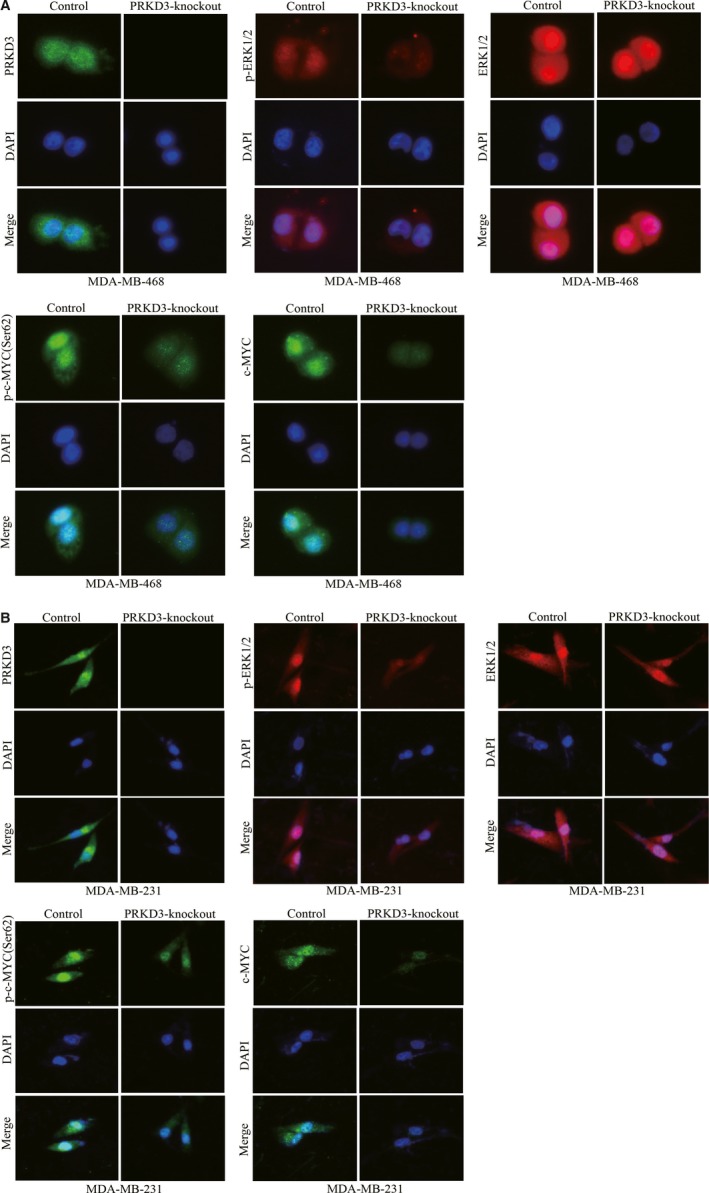
Immunofluorescence staining of PRKD3, (p‐)ERK1/2 and (p‐)c‐MYC in the breast cancer cells. The protein levels of p‐ERK1/2(Thr202/Tyr204), ERK1/2, p‐c‐MYC (Ser62) and c‐MYC in the parental or PRKD3‐knockout (A) MDA‐MB‐468 and (B) MDA‐MB‐231 cells

In addition, Immunofluorescence staining showed that p‐ERK1/2 (Thr202/Tyr204), p‐c‐MYC (Ser62) and c‐MYC were down‐regulated by knocking out PRKD3 in breast cancer cells (Figure 3A,B). These results suggested that PRKD3 likely activates c‐MYC by activating ERK1, but not ERK2.

### 3.3 Loss of PRKD3 decreases c‐MYC target genes expression

**Figure 4:**
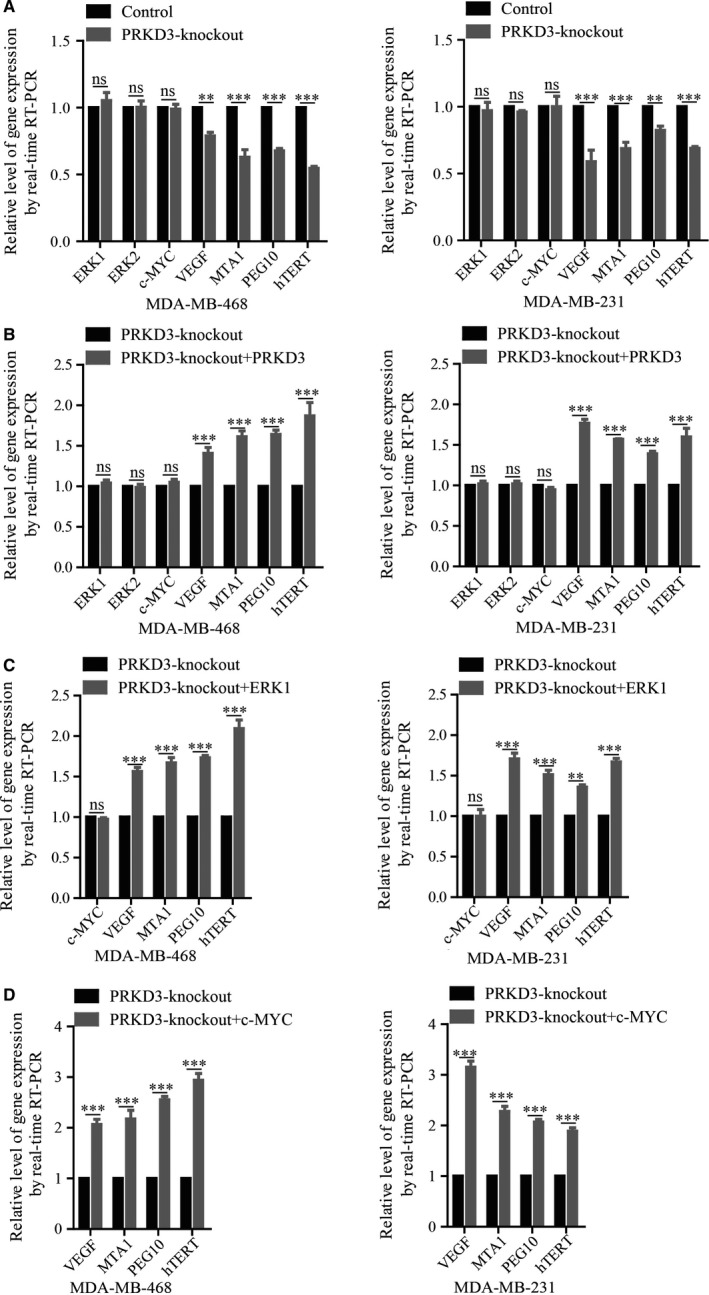
Real‐time RT‐PCR was used to detect the mRNA levels of the PRKD3, ERK1, c‐MYC and the target genes (VEGF, MTA1, PEG10 and hTERT) of c‐MYC. A, The mRNA levels of the genes in the PRKD3‐knockout cells. The mRNA levels of the c‐MYC target genes in the PRKD3‐knockout cell were increased with the ectopic‐ (over)expression of (B) PRKD3, (C) ERK1 or (D) c‐MYC. Data represent the mean ± SEM from three biological replicates (n = 3). **P < .01, and ***P < .001 by *t* test

It was reported that VEGF, MTA1, PEG10 and hTERT were the target genes of c‐MYC. To determine if PRKD3 up‐regulated the expression of the c‐MYC target genes, real‐time RT‐PCR was performed for quantitating the relative amount of the transcripts of the c‐MYC target genes. We found that the mRNA levels of VEGF, MTA1, PEG10 and hTERT in the PRKD3‐knockout breast cancer cells were lower than the ones in the parental cells. Nevertheless, the mRNA levels of ERK1, ERK2 and c‐MYC in the PRKD3‐knockout cells were similar with the ones in the parental cells. (Figure 4A). Additionally, ectopic expression of PRKD3 in the PRKD3‐knockout cells elevated the mRNA levels of VEGF, MTA1, PEG10 and hTERT (Figure 4B). Furthermore, overexpressing ERK1 or c‐MYC in the PRKD3‐knockout cells led to the increased amounts of VEGF, MTA1, PEG10 and hTERT transcripts (Figure 4C,D). These data suggest that PRKD3 up‐regulated the expression of the c‐MYC target genes by activating ERK1/c‐MYC axis but did not up‐regulate the transcription of ERK1 and c‐MYC.

### 3.4 PRKD3/ERK1/c‐MYC pathway promotes breast cancer proliferation

**Figure 5:**
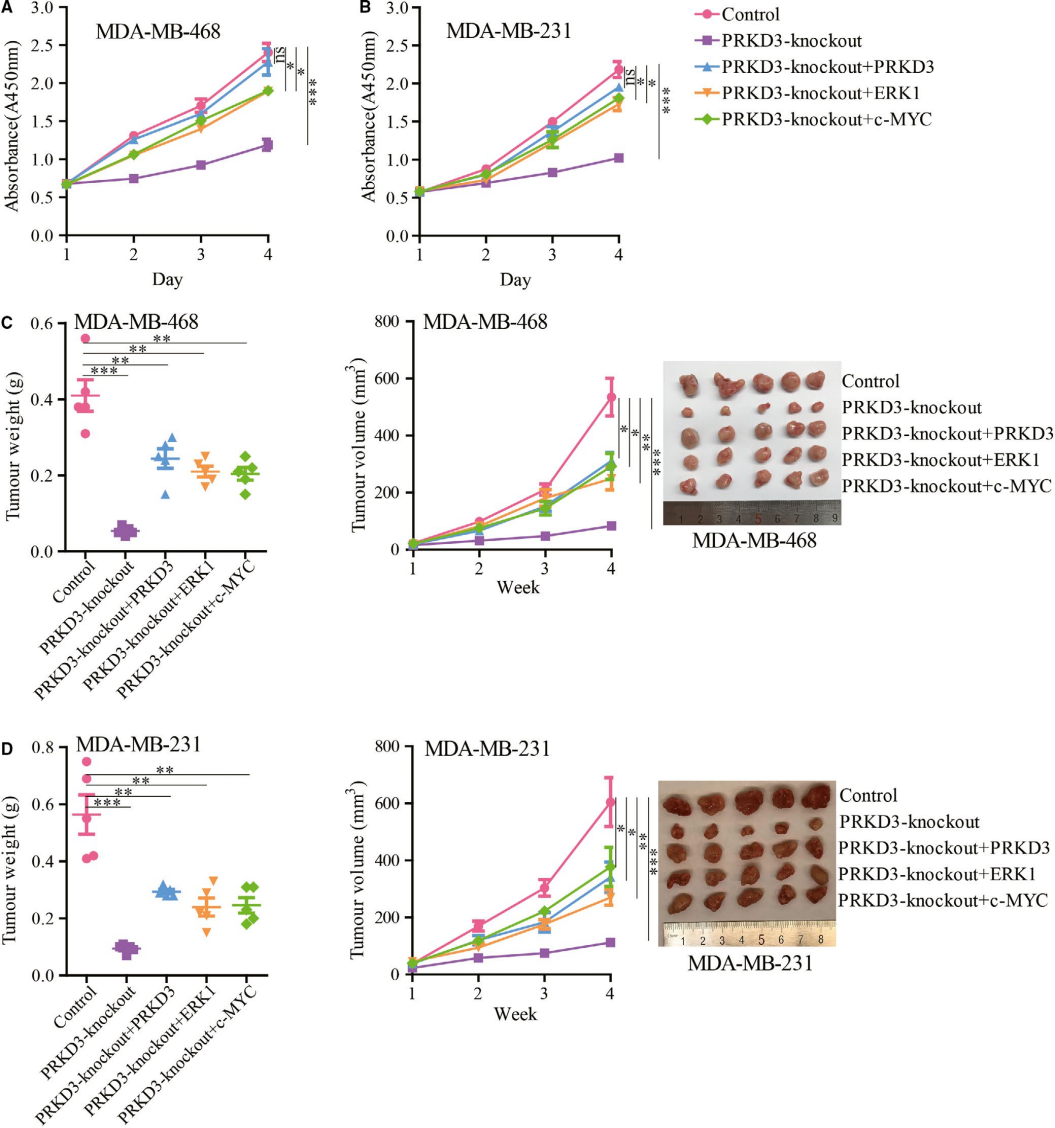
Cell proliferation and xenograft tumour growth measurements using the breast cancer cells. The proliferation of the PRKD3‐knockout (A) MDA‐MB‐468 and (B) MDA‐MB‐231 cells was suppressed. Ectopic (over)expression of PRKD3, ERK1 and c‐MYC in the two cell lines restored the proliferation. The tumour growth inhibition of the PRKD3‐knockout (C) MDA‐MB‐468 and (D) MDA‐MB‐231 cells was shown. Ectopic (over)expression of PRKD3, ERK1 and c‐MYC in the cells restored the tumour growth. Xenografted tumour weight (left), xenografted tumour growth curves (middle), representative xenografted tumour (right) from mouse models. Data represent the mean ± SEM. *P < .05, **P < .01, and ***P < .001 by *t* test

The abovementioned data suggested that PRKD3 mediated the phosphorylation of ERK1 to be activated p‐ERK1, and then the activated p‐ERK1 phosphorylated the Ser62 of c‐MYC to stabilize c‐MYC; as a result, the expression of c‐MYC target genes was up‐regulated by enhancing the stability c‐MYC rather than synthesizing more c‐MYC. To determine if PRKD3/ERK1/c‐MYC pathway promoted breast cancer progression, the cell proliferation assay and tumour formation assay in nude mice were performed. The results of the cell proliferation assay showed that knocking out PRKD3 in the breast cancer cells led to the inhibition of cell proliferation. Additionally, ectopic (over)expression of PRKD3, ERK1 or c‐MYC in the PRKD3‐knockout cells rescued the cell proliferation ability (Figure 5A,B). Consistent with the result of the cell proliferation assay, the volume and weight of the tumour derived from the PRKD3‐knockout cells were significantly smaller and lighter than the ones of the tumour derived from the parental cells (Figure 5C,D). Additionally, the Western blotting data showed that the amounts of p‐ERK1 (Thr202/Tyr204), p‐c‐MYC (Ser62), c‐MYC in the PRKD3‐knockout xenograft tumours were lower than these in the parental xenograft tumours. The amounts of p‐ERK2 (Thr202/Tyr204) and ERK1/2 were not reduced in the PRKD3‐knockout xenograft tumours (Figure S1A,B). Furthermore, the mRNA levels of VEGF, MTA1, PEG10 and hTERT in the PRKD3‐knockout xenograft tumours were lower than that in the parental tumours(Figure S1C,D). Therefore, these results indicated that the PRKD3/ERK1/c‐MYC pathway promoted breast tumour growth in vitro and in vivo.

Taken together, our studies suggested that PRKD3 regulated phosphorylation of ERK1, not ERK2, and then ERK1 stabilized c‐MYC by phosphorylation, leading to promote the proliferation of the breast cancer cells.

## 4 DISCUSSION

In this study, we reported that PRKD3 activated ERK1‐c‐MYC axis to promote the breast tumour growth. The phosphorylation of ERK1 was triggered by an upstream kinase pathway that was mediated by PRKD3 and the stabilized the c‐MYC by the phosphorylation.

Previous studies has reported that the misregulated activation of ERK1/2 and c‐MYC promoted the abnormal cell proliferation in various types of cancers, including breast cancer.^47^, ^48^ This study showed that the loss of PRKD3 restrained the specific phosphorylation of ERK1(thr202/tyr204), but not ERK2 (Figure 2A and Figure 3A,B). Furthermore, epitopic (over)expression of PRKD3 in the PRKD3‐knockout breast cells rescued the phosphorylation of ERK1 (Figure 2B). Our data indicated that the physilogical functions of ERK1 and ERK2 were not totally overlapping in the breast cancer cells. It was reported that activation of p‐ERK (Thr202/Tyr204) enhanced the Ser62 phosphorylation of c‐MYC to stabilize c‐MYC.^19^ Our study showed that the PRKD3‐knockout cells contained the less amount of p‐ERK1 (Thr202/Tyr204) and total c‐MYC than the parental ones (Figure 2A and Figure 3A,B). In addition, ectopic (over)expression of either PRKD3 or ERK1 in the PRKD3‐knockout cells rescued the Ser62 phosphorylation of c‐MYC and the total c‐MYC (Figure 2B,C). We also found that knocking out PRKD3 in the breast cancer cells led to the down‐regulation of the c‐MYC target genes such as VEGF, MTA1, PEG10 and hTERT (Figure 4A) and c‐MYC‐related genes such as CCND, CCNE and E2F (Figure S2). In contrast, ectopic (over)expression of either PRKD3, ERK1 or c‐MYC in the PRKD3‐knockout cells rescued the transcript amounts of the c‐MYC target genes (Figure 4B,C,D). The similar results were also found by siRNA‐induced suppression (Figure S3). The abovementioned results suggested that PRKD3 likely regulated ERK1/c‐MYC axis in the breast cancer cells.

We have reported that PRKD3 was preferentially overexpressed in breast cancer and involved in promoting the breast cancer progression.^2^, ^12^ This study suggested that knocking out PRKD3 led to the inhibition of the breast cancer cell proliferation and tumour growth in vitro and in vivo; however, the ectopic (over)expression of either PRKD3, ERK1 or c‐MYC reversed the inhibition (Figure 5A,B,C and 5). The positive correlation of the transcript levels among PRKD3, ERK1 and c‐MYC in the human breast cancer tissues was not significant, supporting that PRKD3 activated ERK1/c‐MYC axis independent of up‐regulating the transcript levels of the ERK1 and c‐MYC (Figure S4A). Meanwhile, Western blotting showed the proteins level of PRKD3, (p)‐ERK and (p)‐c‐MYC overexpressed in triple‐negative breast cancer tissues compared with paracancerous tissues (Figure S4B). These data indicated that PRKD3 promoted breast cancer proliferation and tumour growth via regulating the ERK1‐c‐MYC axis.

Several previous studies have showed that the protein levels of PRKD3 were higher in breast cancer tissues than in the paracancerous tissues and played an important role in breast cancer promoting processes, suggesting that PRKD3 could be a noval target for developing new therapeutic strategies in breast cancer. Although several pan‐inhibitors have been found to suppress PRKD family activity, such as 2,6‐naphthyridine and bipyridyl inhibitors and their analogs,^49^ 3,5‐diarylazoles,^50^ CID755673 and its analogs,^51^, ^52^ CRT5^53^ and CRT0066101,^10^ and none of them could specifically suppress PRKD3 activity. These inhibitors likely cause many adverse effects in breast cancer patients. Therefore, PRKD3‐specific inhibitors should be developed for the cancer patients.

In short, we suggested that PRKD3 functions as an upstream regulator of ERK1/c‐MYC axis and promotes the proliferation of the breast cancer cells.

## CONFLICT OF INTEREST

All the authors declare that there is no conflict of interest in this manuscript.

## AUTHOR’S CONTRIBUTIONS

YL, SY, JZZ and LMC: involved in study design; YL, HS, SYY, XXM and YHZ: performed the experiments; YL, KHH and LMC: prepared the manuscript; all authors reviewed and approved manuscript.

## Data Availability

The data that support the findings of this study are available from the corresponding author upon reasonable request.
