# Supplementary figures and images for: Protein Kinase D3 promotes the cell proliferation by activating the ERK1/c‐MYC axis in breast cancer
Source: J Cell Mol Med. 2020 Jan 16;24(3):2135–44. doi: 10.1111/jcmm.14772 (PMC7011155; doi:10.1111/jcmm.14772)

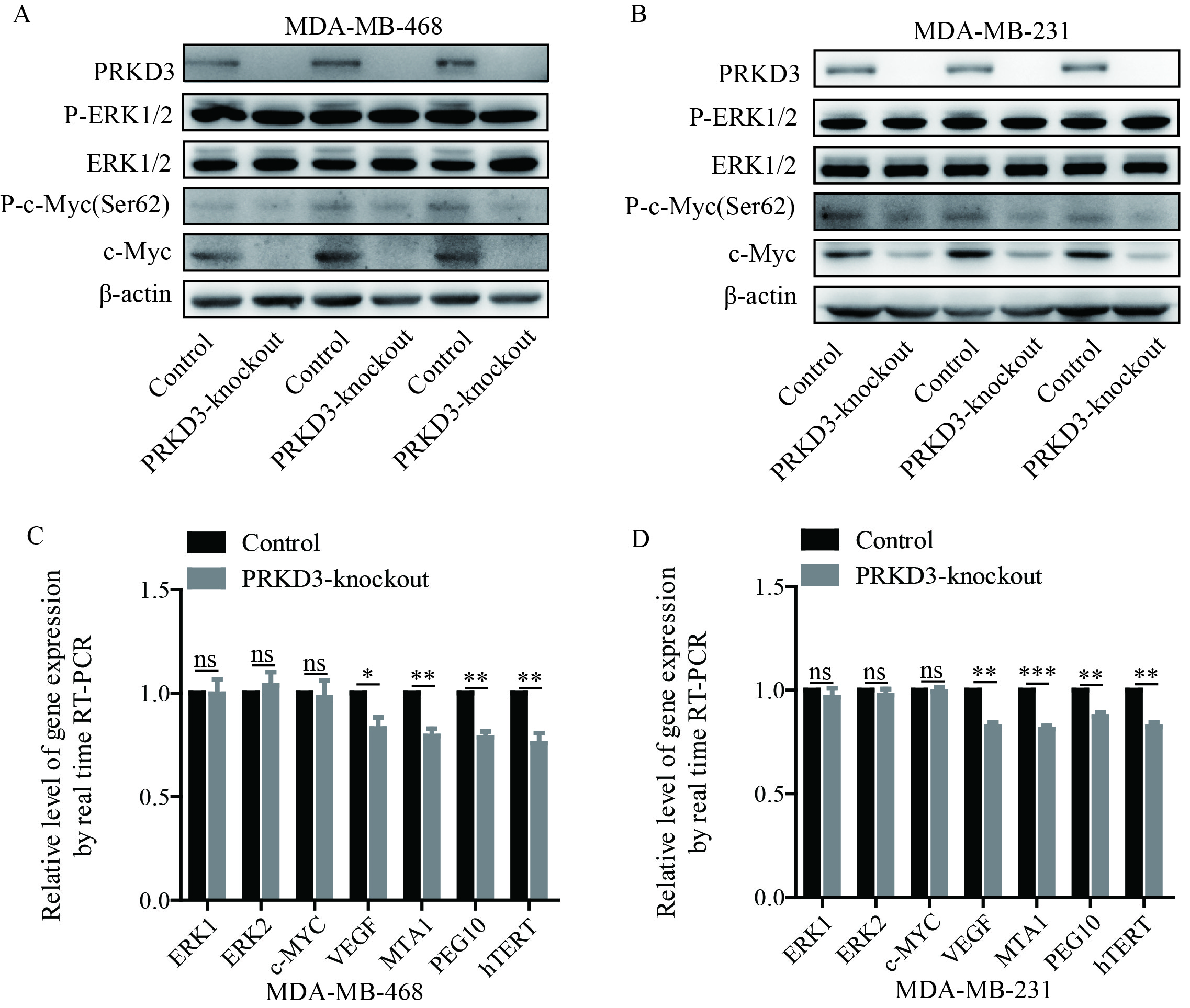

Supplement: Supplementary file 1 [file JCMM-24-2135-s001.jpg]

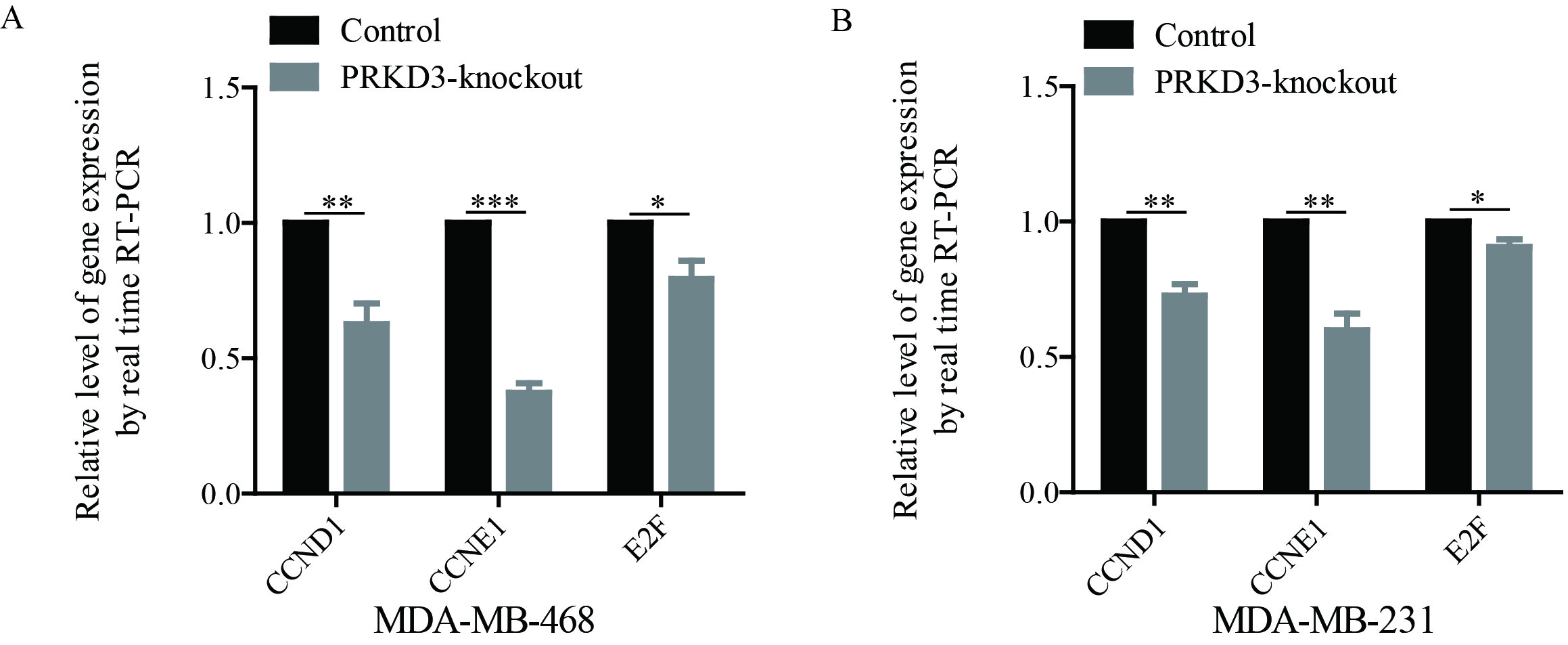

Supplement: Supplementary file 2 [file JCMM-24-2135-s002.jpg]

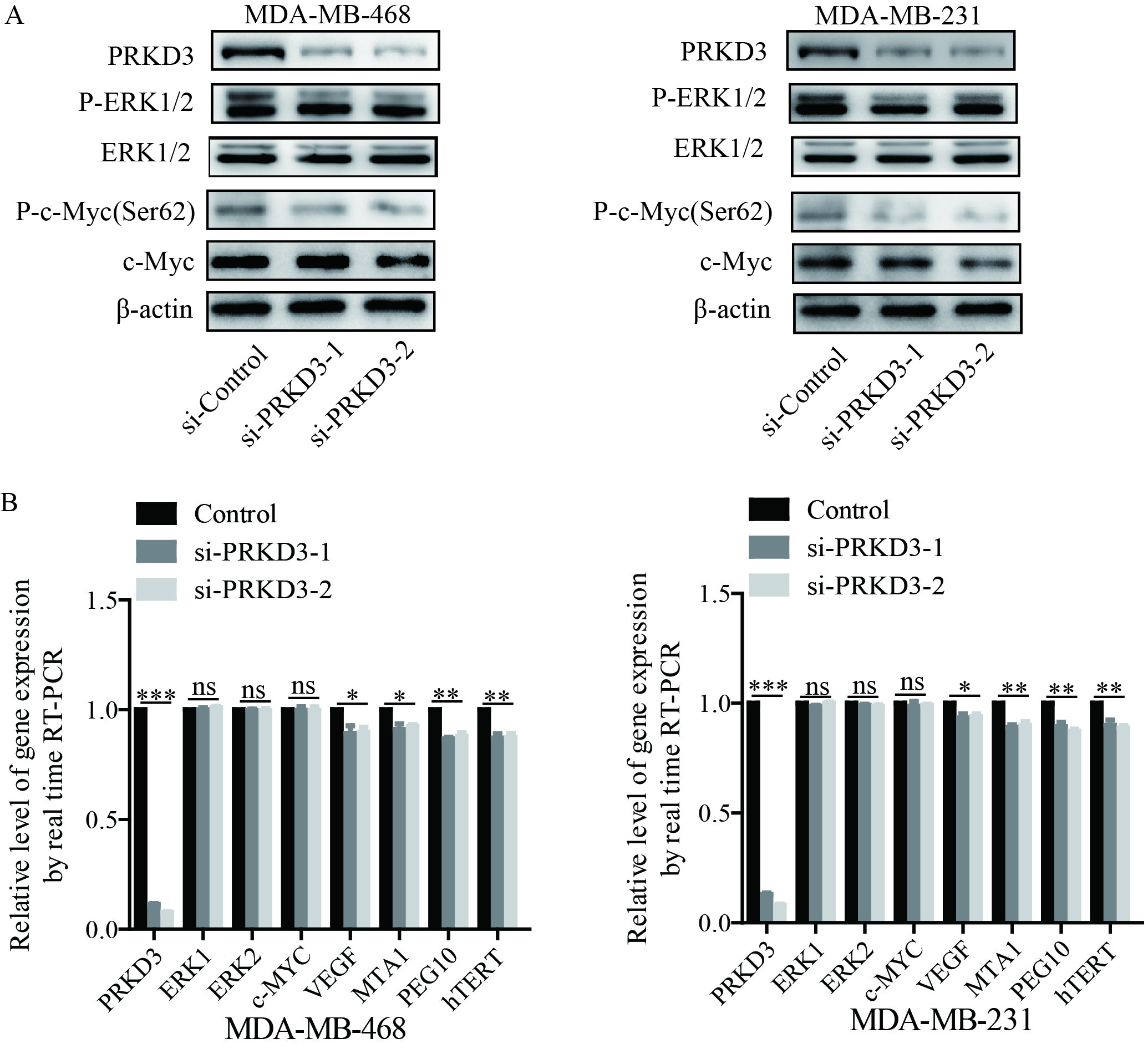

Supplement: Supplementary file 3 [file JCMM-24-2135-s003.jpg]

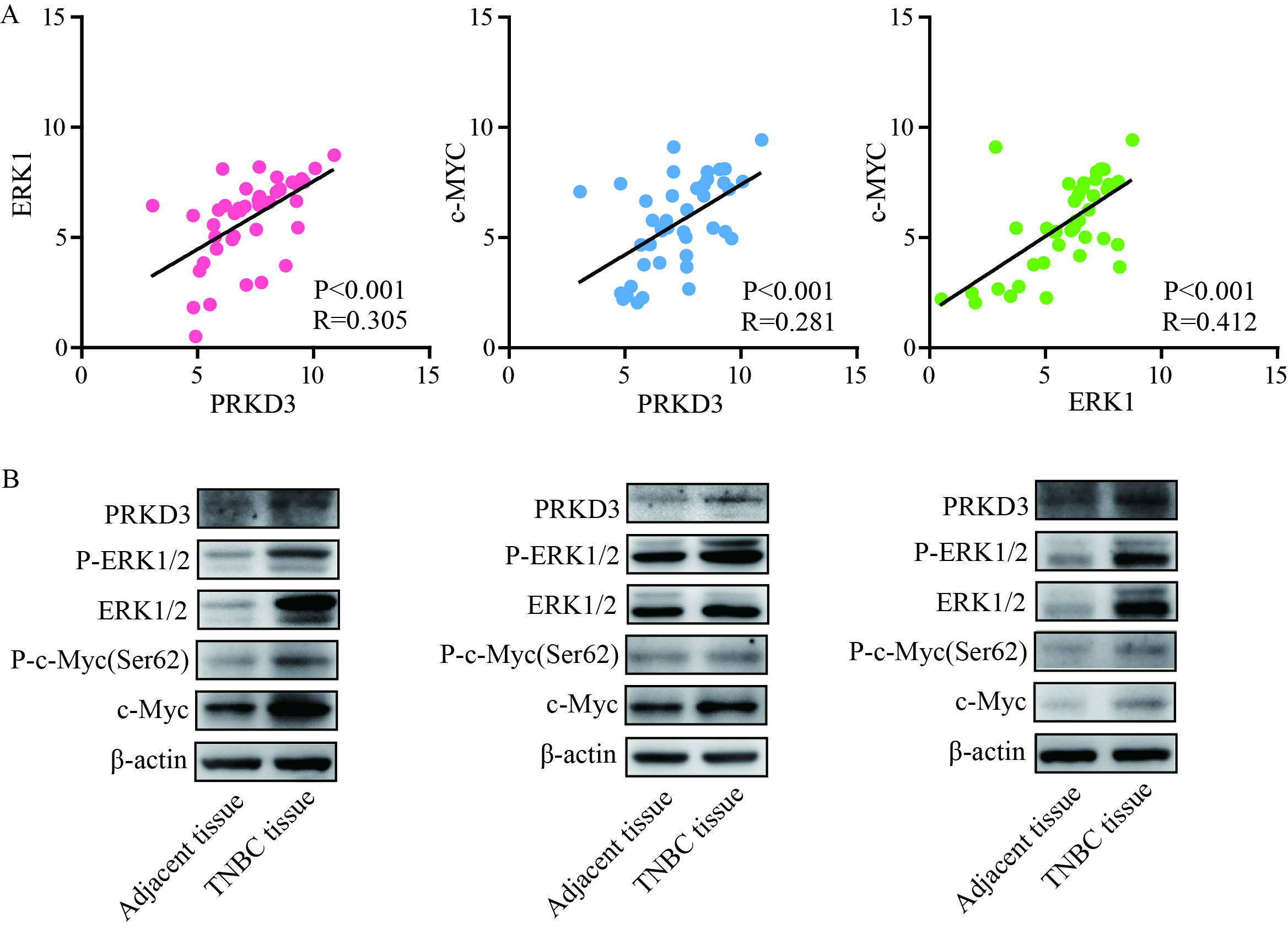

Supplement: Supplementary file 4 [file JCMM-24-2135-s004.jpg]
